# Study rationale and design of the PEOPLHE trial

**DOI:** 10.1007/s11547-024-01764-4

**Published:** 2024-02-06

**Authors:** Gianluca Milanese, Mario Silva, Roberta Eufrasia Ledda, Elisa Iezzi, Chandra Bortolotto, Letizia Antonella Mauro, Adele Valentini, Linda Reali, Olivia Maria Bottinelli, Adriana Ilardi, Antonio Basile, Stefano Palmucci, Lorenzo Preda, Nicola Sverzellati, the PEOPLHE consortium, Lorenzo Aliotta, Sebastiano Barbarino, Santo Borzì, Virginia Casotto, Marco Catalano, Domenico Maria Cavalieri, Mariangela Clemenza, Martina Contino, Luca Crimi, Bruno Curia, Pasquale Favia, Vita Ida Gallone, Giulia Guicciardi, Giuliana La Rosa, Ludovica Leo, Rebecca Mura, Antonella Priore, Lidia Ruongo, Carlotta Scavone, Carlotta Zilioli

**Affiliations:** 1 Unit of Radiological Sciences, University Hospital of Parma, University of Parma, Parma, Italy; 2 University Hospital of Parma, Parma, Italy; 3 Diagnostic Imaging Unit, Department of Clinical, Surgical, Diagnostic, and Pediatric Sciences, University of Pavia, 27100 Pavia, Italy; 4 Radiology Unit—Diagnostic Imaging I, Department of Diagnostic Medicine, Fondazione IRCCS Policlinico San Matteo, Pavia, Italy; 5 Radiology Unit 1, University Hospital Policlinico G. Rodolico-San Marco, Catania, Catania Italy; 6 Department of Medical Surgical Sciences and Advanced Technologies “GF Ingrassia”, University of Catania, University Hospital Policlinico G. Rodolico-San Marco, Catania, Italy; 7 Radiology Unit 1—Department of Medical Surgical Sciences and Advanced Technologies “GF Ingrassia”, University of Catania, University Hospital Policlinico G. Rodolico-San Marco, Catania, Italy; 8 UOSD I.P.T.R.A.—Department of Medical Surgical Sciences and Advanced Technologies “GF Ingrassia”, University of Catania, University Hospital Policlinico G. Rodolico-San Marco, Catania, Italy

**Keywords:** Lung cancer, Lung cancer screening, Low-dose computed tomography, Lung-RADS, Primary and secondary prevention

## Abstract

**Purpose:**

Lung cancer screening (LCS) by low-dose computed tomography (LDCT) demonstrated a 20–40% reduction in lung cancer mortality. National stakeholders and international scientific societies are increasingly endorsing LCS programs, but translating their benefits into practice is rather challenging. The “Model for Optimized Implementation of Early Lung Cancer Detection: Prospective Evaluation Of Preventive Lung HEalth” (PEOPLHE) is an Italian multicentric LCS program aiming at testing LCS feasibility and implementation within the national healthcare system. PEOPLHE is intended to assess (i) strategies to optimize LCS workflow, (ii) radiological quality assurance, and (iii) the need for dedicated resources, including smoking cessation facilities.

**Methods:**

PEOPLHE aims to recruit 1.500 high-risk individuals across three tertiary general hospitals in three different Italian regions that provide comprehensive services to large populations to explore geographic, demographic, and socioeconomic diversities. Screening by LDCT will target current or former (quitting < 10 years) smokers (> 15 cigarettes/day for > 25 years, or > 10 cigarettes/day for > 30 years) aged 50–75 years. Lung nodules will be volumetric measured and classified by a modified PEOPLHE Lung-RADS 1.1 system. Current smokers will be offered smoking cessation support.

**Conclusion:**

The PEOPLHE program will provide information on strategies for screening enrollment and smoking cessation interventions; administrative, organizational, and radiological needs for performing a state-of-the-art LCS; collateral and incidental findings (both pulmonary and extrapulmonary), contributing to the LCS implementation within national healthcare systems.

## Introduction

Lung cancer (LC) is the leading cause of oncologic morbidity and mortality worldwide; accounting for 2.2 million new diagnoses and 1.8 million deaths in 2020 (18% of all cancer deaths) [1]. In Italy, the current prognosis of LC is as poor as 15.9% survival at 5 years after diagnosis [2]. The high mortality rate of LC is related to diagnosis in the advanced stage with limited curative options; hence, control of LC mortality is expected from secondary prevention by early diagnosis with LC screening (LCS), as well as primary prevention by smoking cessation.

Several trials demonstrated 20–40% reduction of LC mortality by low-dose computed tomography (LDCT) screening, which is increasingly endorsed by national stakeholders and international scientific societies [3, 4]. However, the translation of LCS benefits from trial to the general population is challenging, as shown by the US experience where LCS is being reimbursed through Medicare since 2015 [5]. The European Commission is shaping plans to tackle the recognized hurdles of LCS [6]:

- *Engagement of high-risk individuals*, usually nested in the most fragile socioeconomic strata [5, 7].
- *Maintaining high participation rates during the various time points*, which is tackled by the “satisfaction effect” after a first negative screen with consequent drop in the adherence to LCS [8].
- *Continuous quality assurance* for optimization of resources and reduction of risks (related to work up and radiation exposure)

In 2021, the “Model for Optimized Implementation of Early Lung Cancer Detection: Prospective Evaluation Of Preventive Lung HEalth” (PEOPLHE) —an Italian multicentric LCS program—was launched to test the feasibility and implementation of LCS within the national healthcare system. PEOPLHE  addresses specific LCS issues (e.g., enrollment strategies, adherence to LCS rounds, and adherence to smoking cessation programs), testing the feasibility of LCS by sampling three geographically heterogeneous environments through the Italian territory. PEOPLHE is intended to describe:

1. The impact of LCS by assessing strategies to optimize LCS workflow, radiological quality assurance and the need for dedicated resources, including smoking cessation facilities.
2. Quantifying the impact of LCS on life expectancy of high-risk subjects by evaluating standard outcomes, including the proportion of early-stage LC, the number of limited resection surgeries, and surgical approaches performed for benign diseases.

## Material and methods

### Study design

PEOPLHE is a three-year multicentric project supported by the Italian Ministry of Health (MOH) (RF-2019-12371462).

PEOPLHE aims at recruiting 1.500 high-risk individuals across 3 tertiary general hospitals (500 screenees for each center) that are community-based and provide comprehensive services to large populations of three Italian regions, including thoracic oncology multidisciplinary team. The multicentric design aims at exploring geographic, demographic, and socioeconomic diversities that might hamper the implementation of Italian LCS practice.

Selection criteria were derived from the NELSON trial [9], as follows: (i) 50–75 years of age; (ii) smoking habit of > 15 cigarettes/day for > 25 years or > 10 cigarettes/day for > 30 years; (iii) current or former smoker quitting < 10 years; (iiii) no history of cancer in the previous five years.

### Study sites

1. Parma (Emilia Romagna; coordinating center), hereafter called Unit 1: Parma is a medium-sized city (about 200.000 inhabitants) in Northern Italy; its greater area comprises more than 450.000 citizens.
2. Pavia (Lombardy), Unit 2: Pavia is a small/medium-sized city (about 70.000 inhabitants) in Northern Italy; its greater area comprises more than 500.000 citizens.
3. Catania (Sicily), Unit 3: Catania is a medium-sized city (about 300.000 inhabitants), in Southern Italy; its greater area comprises than 1.100.000 citizens.

### Screenees enrollment

PEOPLHE enrollment focuses on a) systematic recruitment of eligible subjects, b) involvement of general practitioners (GP), c) efficiency of referral to LCS hub, and d) capacity needs.

Noteworthy, PEOPLHE was designed before the pandemic of severe acute respiratory distress syndrome coronavirus 2 (SARS-CoV-2), when the peripheral network of GPs was hypothesized to leverage LCS toward the population. However, this structure needed to be converted to cope with the increased workload following the pandemic. Furthermore, tailored approaches are needed to guarantee safe management of patients, screenees and healthcare personnel [10].

The informative campaign rolled out at different levels:

- *Media* level: local and institutional newspapers, social media pages, local television networks on health-dedicated shows. Dedicated informative events were hosted during national anti-tobacco days.
- *Hospital* level: flyers and roll-ups of the PEOPLHE trial reporting study objectives, inclusion criteria, and contacts (email, phone, and QR code toward the subscription website) were placed in the radiology and pneumology departments of the three Units.
- *Healthcare providers* level: dedicated conferences involving local GPs, pulmonologists, and any healthcare provider.

### Administrative and organizational needs

PEOPLHE will assess and quantify the need for dedicated healthcare providers across the full range from recruitment to LC treatment, focusing on radiology department needs, including data managers and administrative staff, radiographers, smoking cessation providers, radiologists, and multidisciplinary teams.

### Smoking cessation

Questionnaires on nicotine addiction will be filled at baseline, at 12 and 24 months, thus recording information on potential variation in the smoking habit over time. The source of information will be the Fagerstrom Test for nicotine dependence. This relatively fast test allows the stratification of four categories of smokers (ranging from low dependence to high dependence).

The PEOPLHE researchers will suggest participation to anti-smoking centres during baseline and recall rounds, providing information on access routes and contacts.

### Radiological quality assurance

The workload of LDCT and multidisciplinary management needs to be defined to estimate the required capacity to deliver a new healthcare service such as LCS.

**Radiological requirements**.

The radiological capacity includes performing CT scanners for low-dose imaging and radiologists experienced in thoracic imaging. The PEOPLHE employs highly performing CT scanners and is coordinated by medical staff with long-standing experience in LCS [11–14].

#### *CT scanning protocols*

CT examinations will be performed according to state-of-the-art technical requirements. Radiation exposure will be maintained as low as reasonably achievable, providing qualitative standards to detect and characterize pulmonary nodules using CT scanners equipped with advanced CT image acquisition systems and radiation dose reduction systems at all Units (e.g., automatic current modulation systems, high sensitivity detectors, iterative reconstruction algorithms); meanwhile, the scanner at Unit 1 will also be equipped with X-ray beam filtration for maximized reduction of radiation exposure (e.g., tin filter). Table 1 details the scanning protocols of each unit.

**Table 1:** Technical information on the CT acquisition protocols of each unit

	Unit 1 (Parma)	Unit 2 (Pavia)	Unit 3 (Catania)
CT scanner	SIEMENS go.TOP	SIEMENS SOMATOM DEFINITION	GENERAL ELECTRIC Optima 660
Tube voltage	Sn100	100	120
Tube current	CARE kV Quality ref. mAs@120 kV = 6	CARE Kv 4D—3 mAs	Noise index 30 (10–40)
Pitch	0.8	1	0.984
Rotation time	0.33	0.5 s	0.5 s
*Reconstruction 1*			
Kernel	Br48	B50f medium sharp	Bone plus
Slice thickness	1 mm	1 mm	0.625 mm
Slice increment	0.7 mm	0.7 mm	0.625–1.250
IR	SAFIRE 3	–	–
Window	Lung	Lung	Lung
*Reconstruction 2*			
Kernel	Sa36	B31f medium smooth +	Detail
Slice thickness	3 mm	3 mm	2.5 mm
Slice increment	1.5 mm	1.5 mm	1.25 mm
IR	SAFIRE 3	–	–
Window	Mediastinum	Mediastinum	Mediastinum
*Reconstruction 3*			
Kernel	Sa36	B31f medium smooth +	Detail
Slice thickness	1 mm	1 mm	1.25 mm
Slice increment	0.7 mm	0.7 mm	0.6–1.25 mm
IR	SAFIRE 3	–	–
Window	Lung	Lung	Bone
CAD software	MM Oncology (SIEMENS Healthineers)	Nodule Detection (Philips Intellispace Portal)	Lung VCAR (General Electric Healthcare)
Volumetry measured on	Reconstruction 3	Reconstruction 3	Reconstruction 3

*SAFIRE* Sinogram affirmed iterative reconstruction

#### *Scheduling LCS*

Trial activities will be held during dedicated LCS time slots: days and daytimes will be tested, and screenees’ preferences recorded.

- Unit 1: different LCS slots will be tested to fulfil participants' preferences (e.g., Monday/Friday afternoon, or Saturday morning).
- Unit 2: dedicated LCS slot will include different LCS slots will be tested to fulfil participants' preferences (e.g., Monday/Friday afternoon).
- Unit 3: dedicated LCS slot will include Friday and Saturday afternoons.

Tailored appointments will be proposed for those screenees for whom participation during the abovementioned LCS slot will not be possible.

The hypothesis behind the organization of dedicated LCS time slots is that it could optimize LCS resources through and homogeneous workflow.

### LDCT reading, reporting and management of nodules

A single radiologist with CAD as second reader will read LDCT scans. Pulmonary nodules will be measured with volumetric approach. LDCT outcome will be based on Lung Imaging Reporting and Data System (Lung-RADS) version 1.1 [15], with biennial round for subjects with negative LDCT, as they were demonstrated to be safe in Italian and Dutch trials and granting a 30% reduction in radiological workload [11, 16–18].

Based on previous LCS data, the screening algorithm will be set on the following LDCT intervals, according to the dominant lesion:

- 24-month interval: Lung-RADS 1 or solid nodule Lung-RADS 2
- 12-month interval: sub-solid nodule included in the dimensional thresholds of Lung-RADS 2
- 6-month interval: solid nodules Lung-RADS 3
- 3-month interval: Lung-RADS 4A
- Work up: Lung-RADS 4B and 4X.

The proposed algorithm will mostly parallel Lung-RADS 1.1 scheme, except for point a) [15].

For CT findings requiring action, management will be based on multidisciplinary management per daily clinical practice.

### Management of collateral and incidental findings

LDCT findings other than pulmonary nodules are classified as:

- “collateral findings”, which include smoking-related abnormalities such as calcifications of coronary arteries, pulmonary emphysema, respiratory bronchiolitis, and interstitial pulmonary abnormality/disease;

*Coronary artery calcification*

Coronary artery calcium (CAC) will be visually scored by a four-point severity scale (0: no CAC; 1: mild CAC—only isolated flecks within a segment; 2: moderate CAC—intermediate CAC between mild and heavy; 3: heavy CAC—continuous CAC within a segment) in each coronary artery (left main, left anterior descending, left circumflex, right coronary artery).

*Emphysema*

Emphysema will be assessed using a semi-quantitative visual scoring method based on a 5-category extent (absent, <5%, 5-25%, 25-50%, > 50%) and will include morphological description (e.g., centrilobular, paraseptal, advanced).

*Interstitial lung abnormality/disease*

Detection of nondependent abnormalities affecting more than 5% of any lung zone identified in subjects without known or suspected interstitial lung diseases (ILD) will be classified as interstitial lung abnormalities (ILA) [19]. Both ILA and ILD will be classified according to current guidelines, including evaluation of distribution (subpleural or non-subpleural), presence of fibrosis (fibrotic or non-fibrotic), and extent (5%-point scale). Frequency, morphology and extent will be recorded, as well as the number of screenees referred to multidisciplinary evaluation.

- “incidental findings”, which encompass non-smoking-related abnormalities [20].

Both collateral and incidental findings will be detailed in the LCS report and managed according to the Quality Assurance Standards prepared for the Targeted Lung Health Checks Programme from the National Health Service (NHS) England. Interventions prompted by detecting these findings on LDCT for LCS will be recorded. The discrepancy between the number of assigned Lung-RADS category “S” and the number of additional investigations will help in defining the cost-efficacy of LCS.

### Data collection and management

Investigators will collect and manage data locally at each site; subsequently, all data will be securely transferred to Unit 1 for quality controls and analysis.

### Scientific output

**Fig. 1:**
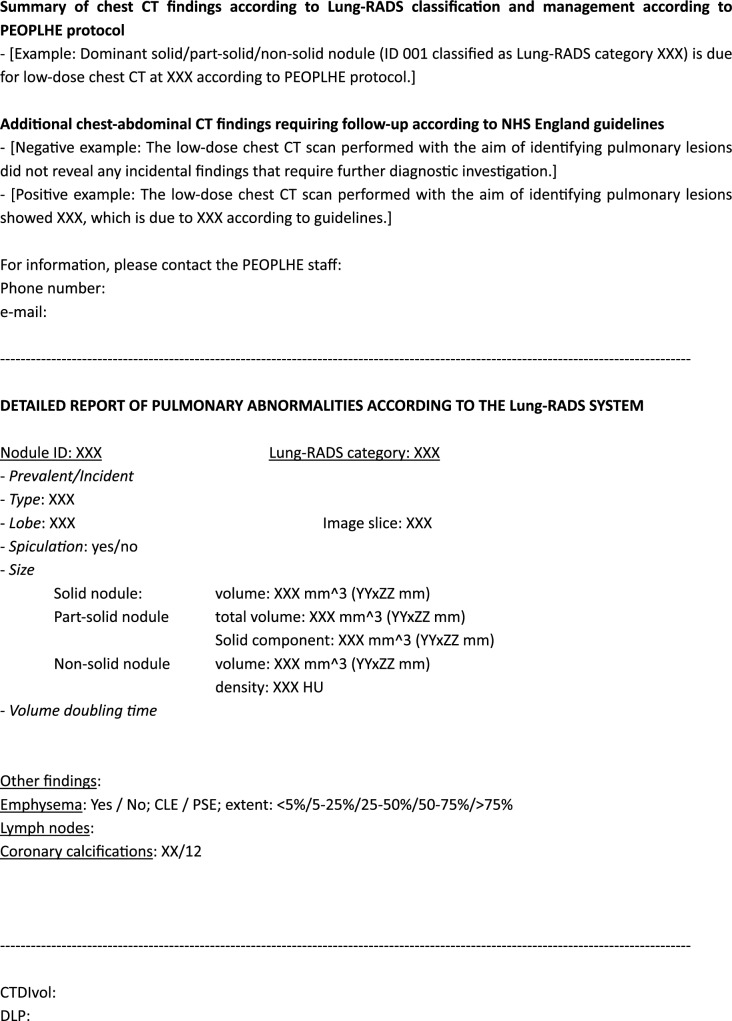
Template of the structured report used within the PEOPLHE project. The first section summarizes the findings: including this information as the first paragraph was intended to increase readability and comprehension for both GPs and screenees; contact details are reported to allow easy interaction with the researchers. Subsequently, we designed a second paragraph including detailed results of LDCT reading (focused on both pulmonary nodules and other findings). The last section of the structured report includes data on radiation exposure and the study protocol.

The first scientific manuscript for the PEOPLHE project will describe the baseline round results, including the strategies used to prompt the enrollment of screenees. Subsequent papers will detail the impact of collateral and incidental findings, and how many diagnostic procedures were requested for findings other than lung cancer (Fig. 1).

### Ethics

The Institutional Review Boards of each Unit approved the study protocol. Before each baseline LDCT, written information on benefits and harms of LCS will be provided and informed consent will be obtained from all screenees. The study was conducted carefully following the indication from Dlgs 101/20 on radiological screening. Participants will be informed about the goals of LCS, along with its potential positive and negative consequences and limitations.

## Discussion

The convincing evidence of LC mortality reduction in large randomized controlled trials ought to European endorsement of LCS implementation. Italy does not currently have a LCS program, but its implementation is being discussed and will be supported by analysis of other ongoing LCS programs [20]. PEOPLHE will address several important issues relevant to the implementation of LCS. It will aim at apportioning the workload and capacity needed for implementation of LCS, including lung cancer screening outcomes and the clinical management of incidental findings. PEOPLHE will also assess the effect, if any, of invitation to LCS has on smoking rates, which was heterogeneous in previous studies and it is known to be subject to bias related to selecting people willing to engage in a research study for health improvement [21–23]. The information from three tertiary Italian hospitals located in different geographical areas is expected to cover a range of geographical and organizational variables.

*Administrative and organizational needs.*

A major LCS challenge is represented by the lack of systematic and coordinated support, especially for a site first approaching LCS activities, which would be the potential situation of almost all Italian hospitals [24]. By reporting the experience of three academic hospitals, we aim to identify the needed up-front infrastructural and manpower investments.

*Enrollment and engagement strategies.*

Identified barriers hampering LCS participation include travel issues or psychological factors (anxiety and stigma), which negatively impact a person’s motivation to attend [25, 26]. Uptake and adherence disparities were potentially related to structural barriers preventing receipt of equitable care, lower socioeconomic status and difficulties in obtaining reimbursements [27–29].

Participation of high-risk populations can be enriched through tailored communication strategies with several invitation approaches, or by offering a mobile setting [30, 31]. The PEOPLHE trial will test the role of telephone triage to present the trial, foster participation of screenees, and organize LCS appointments. A similar approach was previously proposed by the Yorkshire Lung Screening Trial (YLST) in the United Kingdom [7]. Centralization of LDCT in a tertiary hospital is the way to specialized thoracic radiologists and state-of-the-art technology (high-performance scanner, CAD tools, and volumetric nodule measurement). Moreover, the use of mobile CT scanners is associated with higher costs and it seems that scanner location is not the main determinant of LCS uptake [32].

Notably, a minor trust in LCS has been recently reported to challenge LCS implementation in the US, while knowledge of LCS guidelines is a strong and independent predictor for a higher likelihood of suggesting LCS by several healthcare providers [29, 33]. To increase the awareness of the advantages related to LCS, the PEOPLHE staff organized several meetings, both online and in-person, with GPs and other healthcare providers, seeking support from local health associations for recruiting screenees, as well as social media campaigns [34]. A similar role for these health associations has been endorsed by Swiss experts [35].

Previous literature reported generally large rates of drop-out from LCS [36]. We will assess the need for human and financial resources to ensure screenees engagement. Focused analyses of drop-off from LCS based on LDCT outcome categories will be performed, as previous experiences reported higher rates of “no-shows” in subjects with baseline Lung-RADS 3 to 4 as compared with Lung-RADS 1 to 2 [37]. Although potentially affected by the relatively limited time-span of our study, we will explore whether the possibility of low adherence after the baseline round can be counteracted by an NSLT-like active approach, including the administration of annual questionnaires on smoking habit and periodic telephone calls and e-mails.

*Smoking cessation.*

Smoking cessation is the most effective strategy for reducing LC mortality and morbidity. A legitimate concern arose about the possibility that current smokers might consider LCS as a surrogate for an excuse to continue smoking, due to reassurance from a “negative” LDCT. On the other hand, LCS can positively affect smoking cessation among smokers who undergo screening and anti-tobacco counselling [38, 39].

Combined psychological and pharmacologic support have been reported to increase quit rates, with favorable cost-effectiveness ratio when antismoking therapy of natural origin is introduced or when smoking cessation services are proposed immediately after a Target Lung Health Check [14, 40]. However, rates of decline have slowed, and certain categories, namely “hardcore smokers” including smokers less willing to quit, heavy smokers, and who exhibit high-level nicotine dependence, show resistance to tobacco control measures [41–43].

PEOPLHE—by integrating a strict anti-tobacco activity—will record the number of subjects quitting smoking, and the characteristics of both quitters and “hardcore smokers”, expanding the available literature on the topic with potential perspectives on strategies to improve quitting.

*LDCT acquisition, reading and reporting.*

Current recommendations suggest annual LCS throughout a long-time span (up to 25 years) [3] LDCT-based LCS might be associated with the potential risk of radiation-induced LC [44]. Balancing the positive impact of LCS on LC mortality with the risks of radiation exposure is one major focus of LCS literature. Recent improvements in CT hardware and software fostered a great interest in reducing the radiation burden beyond the current state-of-the-art in thoracic imaging (LDCT) toward imaging at a calculated radiation dose of below 1 milliSievert, termed ultra-low-dose CT (ULDCT) [45, 46].

The cost-effectiveness of LCS might vary substantially as it is implemented in real-world settings depending on screenees’ selection, false-positive rate, and rates of invasive procedures. Two independent radiologists have usually performed LCS reading, causing an increased cost for LC [47]. Therefore, the implementation of CAD tools within the workflow can reduce costs and reduce variability in detection rates, with the advantage of reducing the risk of false-negative LDCTs, especially for small pulmonary nodules and nodules in peri-hilar regions [20, 48, 49].

LDCT reports produced in the PEOPLHE trial will be standardized, containing all information bolstered by the European Society of Thoracic Imaging (ESTI) [50]. The PEOPLHE LDCT report has been developed to minimize misunderstandings between screenees and healthcare providers. The outcome and suggested management are clearly stated in the first paragraph, followed by more detailed information; during dedicated phone calls from healthcare providers (trained radiology residents, data managers, experienced radiologists), indeterminate and positive findings and further actions (including the timing of follow-ups or additional investigations) will be discussed.

The PEOPLHE program will provide relevant information on administrative and organizational needs for implementation of lung cancer screening within national healthcare system.
